# Synthesis and anti-nociceptive potential of isoxazole carboxamide derivatives

**DOI:** 10.1186/s13065-019-0518-6

**Published:** 2019-01-29

**Authors:** Hajira Bibi, Humaira Nadeem, Muzaffar Abbas, Muazzam Arif

**Affiliations:** 1 Department of Pharmaceutical Chemistry, Riphah Institute of Pharmaceutical Science, Riphah International University, Islamabad, Pakistan; 2 Department of Pharmacology, Riphah Institute of Pharmaceutical Science, Riphah International University, Islamabad, Pakistan

**Keywords:** Isoxazole, Carboxamide derivatives, Analgesic activity, Cyclooxygenases, Molecular docking

## Abstract

**Background:**

Isoxazole is an important pharmacophore in medicinal chemistry with a wide range of pharmacological activities. The present study deals with the synthesis and evaluation of antinociceptive potential of nine novel 3-substituted-isoxazole-4-carboxamide derivatives.

**Synthesis:**

In the first step, respective oxime was prepared and further treated with ethylacetoacetate and anhydrous zinc chloride followed by hydrolysis of ester to furnish 3-substituted isoxazole-4-carboxylic acid. The respective carboxylic acids were converted to acid chlorides and condensed with aromatic amines to get the target carboxamide derivatives (A1–A5 and B1–B5). These compounds were characterized by FTIR, ^1^HNMR, ^13^CNMR and elemental analysis data and screened for their analgesic activity using acetic acid-induced writhing assay and hot plat test in mice and compared with the standard centrally acting analgesic, tramadol.

**Results:**

All the synthesized carboxamide derivatives showed low to moderate analgesic activity. Among the synthesized derivatives B2 having methoxy (OCH_3_) showed high analgesic activity as compared to tramadol both in acetic acid-induced writhing assay and hot plate assay at dose of 6 mg/kg. To examine the involvement of opioidergic mechanism in the mediation of analgesic effects of isoxazole derivatives animals were further treated with non-selective opioid analgesic, naloxone (0.5 mg/kg). The results showed that compounds A3 and B2 follow a non-opioid receptor pathway in the mediation of analgesic effects. Synthesized compounds A3 and B2 were docked against non-opioid receptors COX-1 (3N8X), COX-2 (1PXX) and human capsaicin receptor (HCR, 3J9J) to analyze their binding interactions. They showed binding energies in the range of − 7.5 to − 9.7 kcal/mol.

**Conclusions:**

The results indicated that isoxazole carboxamide derivatives possess moderate analgesic potential especially compounds A3 and B2 can be considered as lead molecules and explored further for pain management with fewer side effects.

## Introduction

Analgesics selectively relieve pain by acting in the central nervous system (CNS) and/or inhibiting peripheral pain mediators without changing consciousness [1]. Opioid analgesics, nonsteroidal anti-inflammatory drugs (NSAIDS) and local anesthetics are the most widely used analgesic drugs [2]. Traditionally, opioids were considered to exert analgesic effects through actions within the CNS. Recently, however, evidence has begun to accumulate that opioid antinociception can be brought about by activation of opioid receptors (mu, kappa, sigma) located outside the CNS [3]. NSAIDs exert their analgesic effect not only through peripheral inhibition of prostaglandin synthesis but also through a variety of other peripheral and central mechanisms. It is now known that there are two structurally distinct forms of the cyclooxygenase enzyme (COX-l and COX-2). COX-l is a constitutive member of normal cells and COX-2 is induced in inflammatory cells. Inhibition of COX-2 activity represents the most likely mechanism of action for NSAID-mediated analgesia [4].

Isoxazole has broad spectrum of pharmacological activities and also a part of many biodynamic agents due to the presence of an azole with an oxygen atom next to the nitrogen [5]. Isoxazoles nucleus has been used widely in pharmaceutical research and their derivatives are associated with a wide variety of pharmacological properties including hypoglycemic, analgesic, anti-inflammatory, anti-bacterial, anti-HIV, anti-cancer activity, schizophrenia, hypertension and Alzheimer’s disease [6]. Several isoxazole containing molecules make basis for a number of drugs such as sulfisoxazole, sulfamethoxazole, oxacillin, cycloserine. Isoxazoles have extensively been screened for anti-nociceptive activity and possess promising analgesic activity [7–10]. Valdecoxib which is a potent COX-2 inhibitor also contains isoxazole nucleus [11].

The noncovalent binding of a ligand (small molecule) and a receptor (macromolecule) can be anticipated by using computational techniques such as molecular docking studies [12]. The aim of this technique is to find and predict the bound conformation and the binding affinity of ligands [13]. These predictions are of practical significance in drug development in the sense that ligand–protein binding can be used to scrutinize virtual libraries to obtain lead molecules [14]. Furthermore, molecular docking can also be used to assess the bound conformation of known molecules, when the experimental structures are not available [15].

The current study was designed to synthesize and characterize novel 3-substituted isoxazole-4-carboxamide derivatives. The compounds were evaluated for their analgesic potential and related mechanism of anti-nociception in animal models of pain. Molecular docking studies were performed to assess their bound conformations and binding affinities with non-opioid receptors COX-1 and COX-2 and human capsaicin receptor.

## Materials and methods

### Synthesis

#### Synthesis of 3-substituted isoxazole-4-carboxylic acid

Respective aldehyde (0.02 mol) in ethanol was added to aqueous solution of hydroxylamine hydrochloride (0.08 mol) and sodium acetate (0.04 mol). The mixture was heated at 80–90 °C for 30 min. After cooling, the solid separated was collected and purified by recrystallization using ethanol to give corresponding oxime. The oxime (1 mmol) thus obtained was mixed with ethylacetoacetate (2 mmol) and anhydrous zinc chloride (0.1 mmol) in round bottom flask and the contents were gradually heated without any solvent for about an hour. After the completion of reaction (as indicated by TLC), the mixture was cooled to room temperature and ethanol was added with stirring for about 30 min. The resulting solid was treated with 5% NaOH and stirred at room temperature for 4 h. After reaction completion the reaction mixture was acidified with 2 N HCl and the solid separated was recrystallized using ethanol [16].

#### Preparation of 3-substituted isoxazole-4-carboxamide derivatives (A1–A5 and B1–B5)

**Fig. 1:**
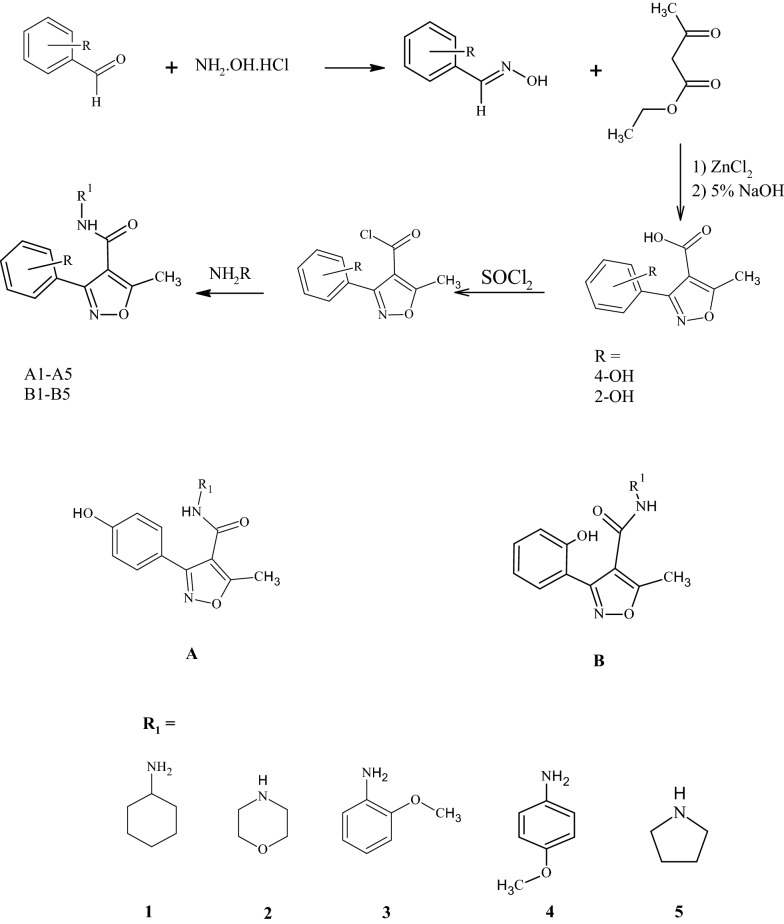
General scheme for the preparation of 3-substituted isoxazole-4-carboxamide

**Fig. 2:**
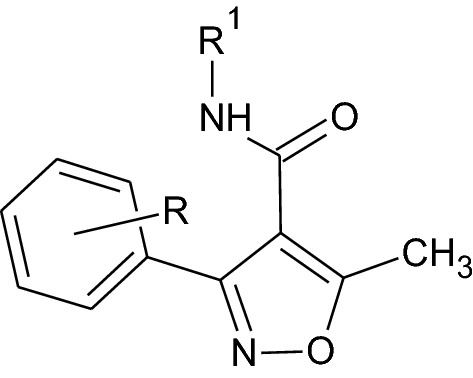
General structure of compounds

The synthesized 3-substituted-isoxazole-4-carboxylic acid from previous step (1 mmol) was refluxed with thionyl chloride (2 mmol) for 2–3 h. After reaction completion, as indicated by TLC, excess liquid was removed under reduced pressure with the caution of not exposing the mixture to air. The resulting acid halide was dissolved in dichloromethane and respective amine (1 mmol) was added to the solution. The reaction mixture was refluxed till completion of reaction as indicated by TLC (ethyl acetate:petroleum ether 1:2). The solvent was removed under reduced pressure and the solid separated was washed with water and recrystallized from ethanol (Figs. 1, 2 and Table 1).

**Table 1:**
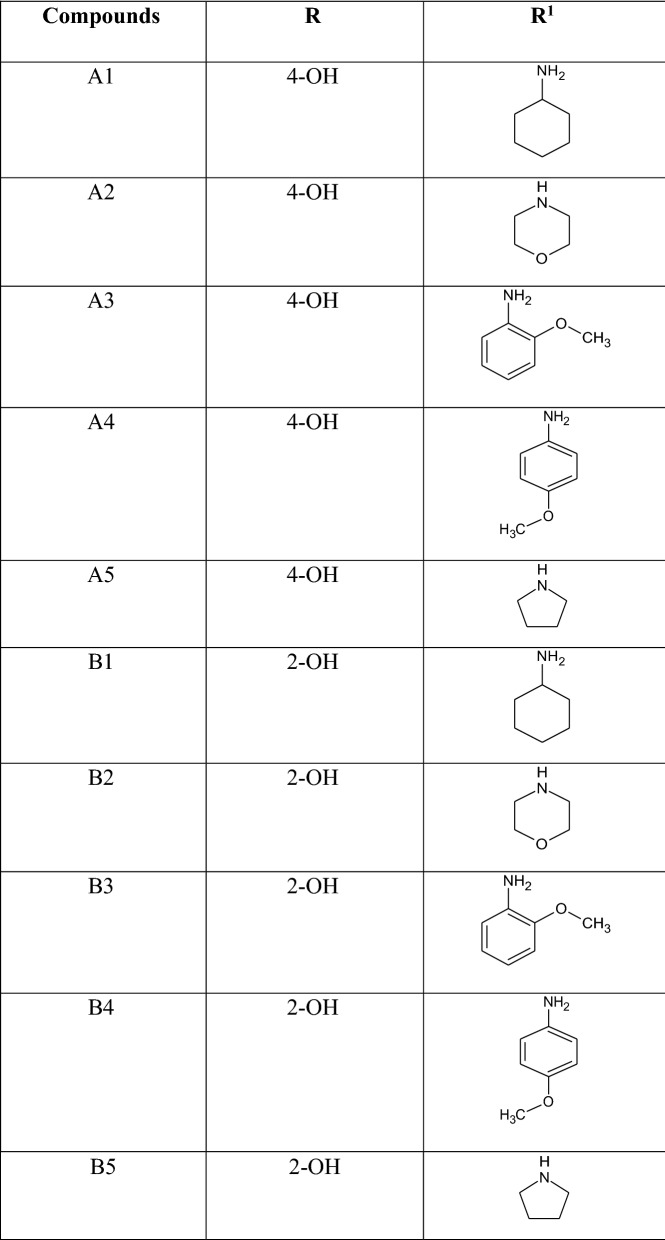
Chemical structure of compound (A1–A5), (B1–B5)

### [3-(4-Hydroxyphenyl)-5-methyl-1,2-oxazol-4-yl](pyrrolidin-1-yl)methanone (A1)

Yield 80%, M.P. = 176 °C, R_f_ = 0.72 (ethylacetate:petroleum ether 1:2), ^1^H NMR: (DMSO, 300 MHz, δ ppm): 1.47–1.74 (m, 10H, J = 13.36 Hz, J = 10.26 Hz, J = 2.79 Hz, cyclohexyl-H), 3.86 (s, 1H, cyclohexyle-H), 7.20–7.63 (m, 4H, J = 8.8 Hz, J = 1.27 Hz, J = 0.46 Hz, Ar–H), 2.63 (s, 3H, CH_3_ isoxazole). ^13^CNMR (DMSO-d_6_, 100 MHz, δ ppm): 156.2, 114.8, 124.5, 128.9 (Ar–C), 159.8, 175.1, 110.3 (isoxazole-C), 160.2 (CONH), 11.3 (isoxazole-CH_3_), 48.5, 33.9, 24.8, 24.8 (cyclohexyl-C). IR (cm^−1^): 3400 (N–H), 3000 (sp^2^CH), 1660 (C=C), 1665 (C=N), 1650 (C=O), 3295 (O–H). Anal. Calcd. For C_17_H_20_N_2_O_3_: C, 68.00; N, 9.33; O, 16.00. Found: C, 67.97; N, 9.30; O, 16.04.

### [3-(4-Hydroxyphenyl)-5-methyl-1,2-oxazol-4-yl](morpholin-4-yl)methanone (A2)

Yield 50%, M.P. = 190 °C, R_f_ = 0.4 (ethylacetate:petroleum ether 1:2), ^1^H NMR: (DMSO, 300 MHz, δ ppm), 7.25–7.52 (m, 4H, J = 12.9 Hz, J = 9.1 Hz, J = 2.02 Hz, Ar–H), 2.60 (s, 3H, CH_3_ isoxazole), 3.61–3.62 (m, 8H, J = 15.16 Hz, J = 3.27 Hz, J = 12.24 Hz morpholine-H). ^13^CNMR (DMSO-d_6_, 100 MHz, δ ppm): 157.0, 115.3, 125.6, 127.9 (Ar–C), 159.3, 172.3, 111.3 (isoxazole-C), 160.9 (CONH), 11.9 (isoxazole-CH_3_), 44.5, 66.9 (morpholine-C). IR (cm^−1^): 3100 (N–H), 1690 (C=C), 1679 (C=N), 1708 (C=O), 3324 (O–H). Anal. Calcd. For C_15_H_16_N_2_O_4_: C, 62.50; N, 9.72; O, 22.22. Found: C, 62.51; N, 9.70; O, 22.19.

### 3-(4-Hydroxyphenyl)-*N*-(2-methoxyphenyl)-5-methyl-1,2-oxazole-4-carboxamide (A3)

Yield 65%, M.P. = 170 °C, R_f_ = 0.65 (ethylacetate:petroleum ether 1:2), ^1^H NMR: (DMSO, 300 MHz, δ ppm): 6.50–7.65 (m, 8H, J = 8.4 Hz, J = 9.0 Hz, Ar–H), 3.77 (s, 3H, OCH_3_), 3.38 (s, 3H, CH_3_ isoxazole). ^13^CNMR (DMSO-d_6_, 100 MHz, δ ppm): 155.8, 115.9, 127.5, 128.9 (Ar–C), 158.0, 173.7, 112.5 (isoxazole-C), 162.9 (CONH), 13.0 (isoxazole-CH_3_), 111.9, 120.9, 122.0, 123.9, 124.9, 149.3 (phenyl-C) 56.7 (phenyl OCH_3_). FTIR (cm^−1^): 3440 (N–H), 1675 (C=N), 1635 (C=C), 1725 (C=O), 3450 (O–H). Anal. Calcd. For C_18_H_16_N_2_O_4_: C, 66.67; N, 8.64; O, 19.75. Found: C, 66.73; N, 8.68; O, 19.73.

### 3-(4-Hydroxyphenyl)-*N*-(4-methoxyphenyl)-5-methyl-1,2-oxazole-4-carboxamide (A4)

Yield 55%, M.P. = 205 °C, R_f_ = 0.8 (ethylacetate:petroleum ether),^1^H NMR: (DMSO, 300 MHz, δ ppm): 6.53–7.33 (m, 8H, J = 8.86 Hz, J = 1.26 Hz, J = 0.46 Hz, Ar–H), 3.66 (s, 3H, OCH_3_), 2.59 (s, 3H CH_3_ isoxazole). ^13^CNMR (DMSO-d_6_, 100 MHz, δ ppm): 158.3, 113.7, 127.9, 129.3 (Ar–C), 159.6, 174.4, 113.4 (isoxazole-C), 164.1 (CONH), 11.8 (isoxazole-CH_3_), 113.8, 121.8, 128.3, 154.7 (phenyl-C) 55.1 (phenyl OCH_3_). IR (cm^−1^): 3035 (N–H), 1350 (sp3CH), 1660 (C=N), 1635 (C=C), 1728 (C=O), 3415 (O–H). Anal. Calcd. For C_18_H_16_N_2_O_4_: C, 66.67; N, 8.64; O, 19.75. Found: C, 66.70; N, 8.63; O, 19.79.

### 3-(4-Hydroxyphenyl)-*N*-(4-methoxyphenyl)-5-methyl-1,2-oxazole-4-carboxamide (A5)

Couldn’t be isolated.

### [3-(2-Hydroxyphenyl)-5-methyl-1,2-oxazol-4-yl](pyrrolidin-1-yl)methanone (B1)

Yield 89%, M.P. = 220 °C, R_f_ = 0.75 (ethylacetate:petroleum ether), ^1^H NMR: (DMSO, 300 MHz, δ ppm): 7.10–7.22 (m, 8H, J = 8.01 Hz, J = 7.61 Hz, J = 1.09 Hz, Ar–H), 3.76 (s, 1H, cyclohexyl-H), 1.37–1.64 (m, 10H, J = 12.29 Hz, J = 2.79 Hz, J = 10.26 Hz, cyclohexyl-H), 2.59 (s, 3H, CH_3_ isoxazole). ^13^CNMR (DMSO-d_6_, 100 MHz, δ ppm): 155.6, 116.7, 130.6, 118.1, 127.3, 121.3 (Ar–C), 156.7, 172.9, 113.7 (isoxazole-C), 163.1 (CONH), 12.6 (isoxazole-CH_3_), 46.7, 32.6, 24.8, 25.7 (cyclohexyl-C). IR (cm^−1^): 3300 (N–H), 3358 (O–H), 1730 (C=O), 1650 (C=N), 1680 (C=C). Anal. Calcd. For C_17_H_20_N_2_O_3_: C, 68.00; N, 9.33; O, 16.00. Found: C, 68.04; N, 9.36; O, 15.96.

### [3-(2-Hydroxyphenyl)-5-methyl-1,2-oxazol-4-yl](morpholin-4-yl)methanone (B2)

Yield 75%, M.P. = 210 °C, R_f_ = 0.55 (ethylacetate:petroleum ether), ^1^H NMR: (DMSO, 300 MHz, δ ppm): 6.62–7.07 (m, 4H, J = 10.26 Hz, J = 9.1 Hz, Ar–H), 3.32–3.53 (m, 8H, J = 15.2 Hz, J = 3.32 Hz, J = 2.4 Hz, morpholine-H), 3.77 (s, 3H, CH_3_ isoxazole). ^13^CNMR (DMSO-d_6_, 100 MHz, δ ppm): 155.9, 116.7, 132.6, 117.2, 129.0, 121.1 (Ar–C), 156.9, 174.6, 112.7 (isoxazole-C), 163.2 (CONH), 12.9 (isoxazole-CH_3_), 43.2, 65.7 (morpholine-C). FTIR (cm^−1^): 3400 (N–H), 1670 (C=C), 1660 (C=N), 1718 (C=O), 3430 (O–H). Anal. Calcd. For C_15_H_16_N_2_O_4_: C, 62.50; N, 9.72; O, 22.22. Found: C, 62.47; N, 9.73; O, 22.27.

### 3-(2-Hydroxyphenyl)-*N*-(2-methoxyphenyl)-5-methyl-1,2-oxazole-4-carboxamide (B3)

Yield 60%, M.P. = 195 °C, R_f_ = 0.62 (ethylacetate:petroleum ether),^1^H NMR: (DMSO, 300 MHz, δ ppm): 7.13–7.20 (m, 8H, J = 8.01 Hz, J = 1.67 Hz, J = 0.46 Hz, Ar–H), 3.70 (s, 3H, OCH_3_), 2.64 (s, 3H, CH_3_ isoxazole). ^13^CNMR (DMSO-d_6_, 100 MHz, δ ppm): 157.6, 116.7, 130.5, 119.3, 127.6, 121.9 (Ar–C), 155.3, 175.1, 110.1 (isoxazole-C), 160.2 (CONH), 12.6 (isoxazole-CH_3_), 112.3, 123.7, 124.5, 120.3, 121.8, 146.4 (phenyl-C) 55.7 (phenyl OCH_3_). IR (cm^−1^): 3400 (N–H), 1682 (C=N), 1665 (C=C), 1710 (C=O), 3325 (O–H). Anal. Calcd. For C_18_H_16_N_2_O_4_: C, 66.67; N, 8.64; O, 19.75. Found: C, 66.62; N, 8.60; O, 19.77.

### 3-(2-Hydroxyphenyl)-*N*-(4-methoxyphenyl)-5-methyl-1,2-oxazole-4-carboxamide (B4)

Yield 60%, M.P. = 225 °C, R_f_ = 0.89 (ethylacetate:petroleum ether), ^1^H NMR:(DMSO, 300 MHz, δ ppm): 6.74–7.46 (m, 8H, J = 8.01 Hz, J = 0.46 Hz, J = 1.60 Hz, Ar–H), 3.77 (s, 3H, OCH_3_), 3.38 (s, 3H, CH_3_ isoxazole). ^13^CNMR (DMSO-d_6_, 100 MHz, δ ppm): 157.2, 115.4, 117.5, 121.5, 130.1, 128.9 (Ar–C), 157.3, 172.7, 111.3 (isoxazole-C), 163.2 (CONH), 13.3 (isoxazole-CH_3_), 114.3, 122.6, 126.8, 155.1 (phenyl-C) 57.3 (phenyl OCH_3_). IR (cm^−1^):1703 (C=O), 3100 (N–H), 1630 (C=C), 1665 (C=N), 3410 (O–H). Anal. Calcd. For C_18_H_16_N_2_O_4_: C, 66.67; N, 8.64; O, 19.75. Found: C, 66.69; N, 8.66; O, 19.71.

### [3-(2-Hydroxyphenyl)-5-methyl-1,2-oxazol-4-yl](pyrrolidin-1-yl)methanone (B5)

Yield 25%, M.P. = 215 °C, R_f_ = 0.5 (ethylacetate:petroleum ether),^1^H NMR: (DMSO, 300 MHz, δ ppm): 6.67 (d, 2H, J = 8.2 Hz, Ar–H), 2.9–3.34 (m, 8H, pyrrolidine-H), 2.98 (s, 3H, CH_3_ isoxazole). ^13^CNMR (DMSO-d_6_, 100 MHz, δ ppm): 157.7, 116.3, 119.4, 121.7, 127.1, 131.3 (Ar–C), 155.9, 172.8, 111.9 (isoxazole-C), 161.9 (CONH), 12.9 (isoxazole-CH_3_), 24.4, 26.1, 47.3 (pyrrolidine-C). IR (cm^−1^): 3300 (N–H), 1731 (C=O), 1660 (C=N), 1680 (C=C), 3290 (O–H). Anal. Calcd. For C_15_H_16_N_2_O_3_: C, 66.17; N, 10.29; O, 17.64. Found: C, 66.22; N, 10.31; O, 17.62.

### Analgesic activity

Analgesic activity of compounds A1–A5 and B1–B5 was performed by two models i.e.,

- Acetic acid—mediated writhings.
- Hot plate assay.

#### Acetic acid-induced writhing test

The anti-nociceptive potential of compounds A1–A5 and B1–B5 was determined by using acetic acid-induced writhing test. Briefly, mice were fasted for 2 h before performing the activity and were divided into four groups. Group 1 was used as negative control (saline group, 10 mL/kg, i.p.), group 2 and 3 was used for dose of each compound (6 and 3 mg/kg, i.p.), while group 4 was assigned as positive control (tramadol, 3 mg/kg, i.p.). Mice were injected test compound and standard drug. Pain was induced in mice after 30 min by intraperitonial injection of 1% acetic acid (10 mL/kg). The mice were placed individually in transparent cage. The numbers of acid-induced writhes (abdominal stretches and/or simultaneous stretching of one hind limb) were counted for 20 min [17].

#### Hot plate assay

Hot plate assay was performed for the determination of central anti-nociceptive activity of compounds A1–A5 and B1–B5. Food was withdrawn 2 h before performing this assay. Mice were divided into four groups. Group 1 is used as negative control (saline group, 10 mL/kg, i.p.), group 2 and 3 was used for dose of each compound (6 and 3 mg/kg, i.p.), while group 4 was assigned as positive control (tramadol, 3 mg/kg, i.p.). Mice were injected respective compound and standard drug. Thirty minutes after drug administration, mice were placed on hot plate maintained at 55 °C and latency to a pain reaction (licking, flicking of rear paw or jumping) was recorded in seconds, assessing their response to thermal stimulus. Cut off time was 30 s to avoid thermal par injury. The reaction time was taken at 0, 30, 60 and 120 min after administration of treatment. The latency time in seconds was compared to that of test drug treated animals [18].

### Docking studies

Molecular docking is used to investigate the affinity between ligand and protein targets. We used AutoDock Vina program for docking study through PyRx [7]. Affinity of best docked pose of ligand and protein target complex was determined by E-value (Kcal/mol). It provides prediction of binding free energy and binding constant for docked ligands [9]. 3D-structures of test compounds (A1–A5, B1–B5) were prepared in Discovery Studio Visualizer (DSV) and saved as PDB format. 3D-structures of target proteins were taken from http://www.rcsb.org/pdb/home/home.do. The target proteins involved in pain pathways are cyclooxygenase-1 (COX-1, PDB-ID: 3N8X), cyclooxygenase-2 (COX-2 PDB-ID: 1PXX) and human capsaicin receptor (HCR, PDB-ID: 3J9J). These target proteins were then purified by Biovia Discovery Studio Client 2016. Both test compounds along with protein targets were loaded in a software named as PyRx and then docked against respective targets. Binding affinity was calculated shown in Kcal/mol. For post docking interactions Discovery Studio Visualizer was used for number of Hydrogen bonds and binding amino acid residue: alanine (ALA), glutamic acid (GLU), asparagine (ASN), arginine (ARG), aspartic acid (ASP), lysine (LYS), serine (SER), threonine (THR) etc.

### Data analysis

Data were presented as mean ± SEM and were analyzed through one-way ANOVA followed by Tukey’s post hoc test using Graph Pad Prism 6.0. A P < 0.05 will be considered significant.

## Results and discussion

### Chemistry

All the synthesized compounds were characterized by FTIR and ^1^HNMR. All the synthesized compounds were obtained in the solid form. Molecular weights were in the range of 272 to 324 g/mol and their melting points were above 170 °C and most of them decomposed at that temperature. Yield of all compounds were above 50%.

The formation of final products was confirmed by ^1^HNMR spectral data. Aromatic protons appeared as multiplets in the range of 6.50–7.69 ppm for all the compounds. In case of A1 and B1, cyclohexylamine protons resonated as multiplet at 1.37–1.64 ppm and 1.47–1.74 ppm respectively. In compounds having morpholine, a multiplet due to morpholine protons appeared at 3.51–3.62 ppm which confirmed the final products. In case of B5, a multiplet was observed at 2.9–3.34 ppm due to pyrrolidine ring. In case of A4 and B4, a triplet due to OCH_3_ group was appeared at 3.66 ppm and 3.77 ppm respectively. The singlet of CH_3_ protons of isoxazole was appeared in the range 2.59–3.38 ppm in all compounds. The synthesis of these compounds was also confirmed by their ^13^CNMR spectral data. The carbon of amide linkage showed its peaks at 162–160 ppm in all compounds. Peaks for aromatic carbon appeared at 157–113 ppm. The methyl carbon of isoxazole ring resonated at 11–10 ppm.

### Analgesic activity of synthesized derivatives

All the synthesized compounds were evaluated for analgesic activity but most of them showed no decrease in writhes and latency time both in acetic acid-induced writhing test and hot plate test i.e., A1, A2, A4, B1, B3, B4, B5. They remained inactive at the dose (6 mg/kg) when compared with standard analgesic, tramadol (3 mg/kg). Synthesized compounds A3 and B2 showed analgesic activity in these assays. The maximum writhes count of A3 treated group (6 mg/kg) decreased to 26 ± 2.28 (P < 0.001 vs. saline group). This might be due to the presence of *p*-anisidine moiety in these compounds. Tramadol (3 mg/kg) decreased numbers of writhes to 25.25 ± 0.62 (P < 0.001 vs. saline group). The maximum writhes count of B2 treated group (6 mg/kg) decreased to 7.75 ± 3.19 (P < 0.001 vs. saline group) and it showed a potent analgesic effect having OCH_3_ group at ortho position. Animal were treated with non-selective opioid antagonist naloxone (0.5 mg/kg) to examine the involvement of opioidergic mechanism in the mediation of analgesic effects of these compounds. The results showed that naloxone did not reverse the analgesic effect these compounds indicating A3 and B2 follow a non-opioid receptor pathway to decrease the pain in animal models. Conclusively, A3 and B2 showed low to moderate analgesic effect.

**Fig. 3:**
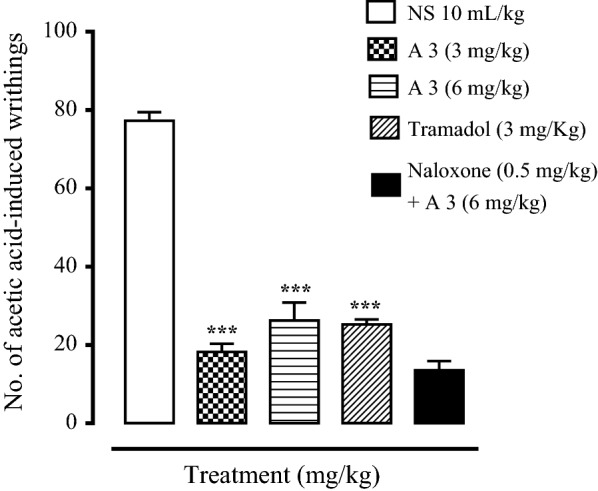
Effect of A3, tramadol and naloxone on acetic acid-induced writhes in mice. Values are shown as mean ± SEM, n = 4. ***P < 0.001 vs. saline group, one-way ANOVA with post hoc Tukey’s test

**Fig. 4:**
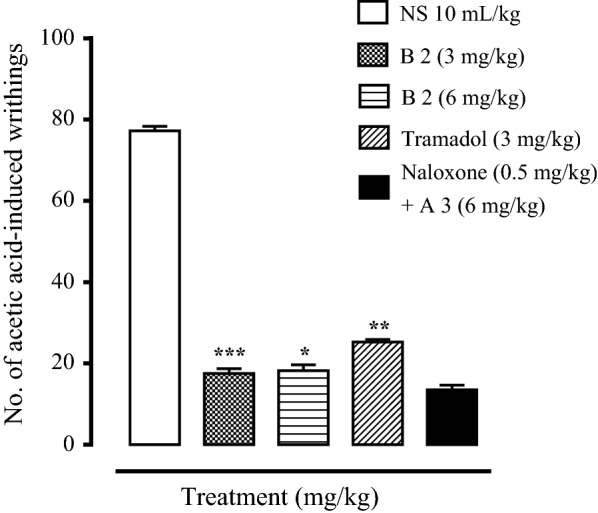
Effect of B2, tramadol and naloxone on acetic acid-induced writhes in mice. Values are shown as mean ± SEM, n = 4. ***P < 0.001 vs. saline group, one-way ANOVA with post hoc Tukey’s test

**Fig. 5:**
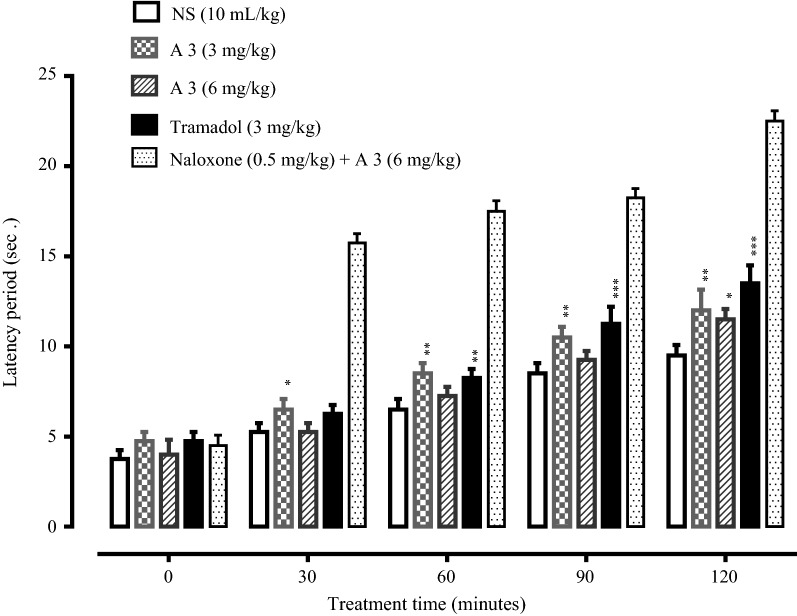
Effect of A3, tramadol and naloxone on latency time in hot plate assay. Values are expressed as mean ± SEM, n = 4. ***P < 0.001 vs. saline group, one-way ANOVA with post hoc Tukey’s test

**Fig. 6:**
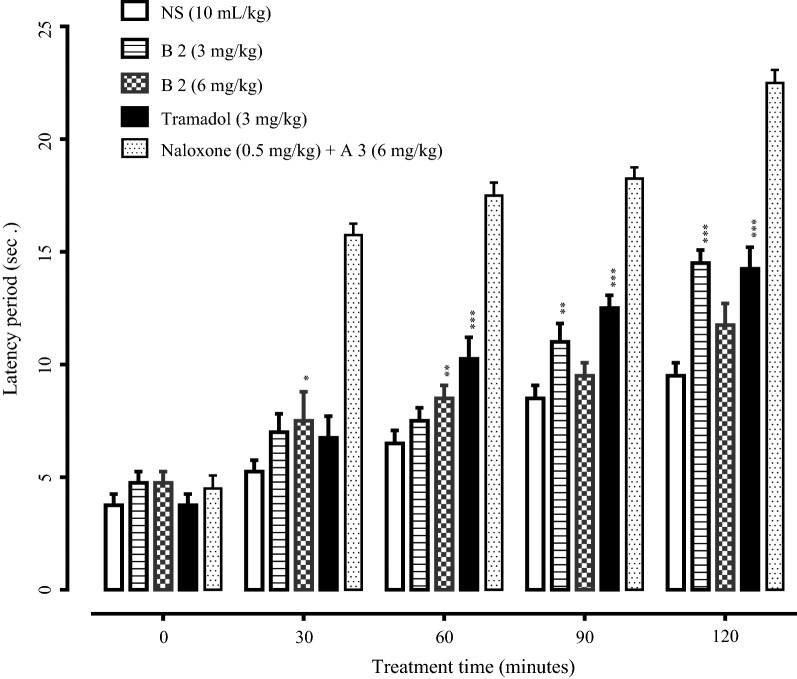
Effect of B2, tramadol and naloxone on latency time in hot plate assay. Values are expressed as mean ± SEM, n = 4. ***P < 0.001 vs. saline group, one-way ANOVA with post hoc Tukey’s test

Hot plate assay was also performed to investigate the latency time of all the synthesized compounds. Tramadol was used as a reference standard. Compounds A1, A2, A4, B1, B3, B4, B5 showed no significant increase in latency time. While compounds A3 and B2 showed good latency time compared with tramadol. Compound A3 (3 mg/kg) showed maximum latency time. The latency time of compound A3 (3 mg/kg) treated group at 0, 30, 60, 90 and 120 min was 4.75 ± 0.25, 6.50 ± 0.28, 8.50 ± 0.28, 10.50 ± 0.28 and 12.00 ± 0.57 s (P < 0.001 vs. saline group) respectively. The latency time of compound B2 (3 mg/kg) treated group at 0, 30, 60, 90 and 120 min was 4.75 ± 0.25, 7.00 ± 0.40, 7.50 ± 0.28, 11.00 ± 0.40 and 14.50 ± 0.28 s (P < 0.001 vs. saline group respectively. The latency time of tramadol (3 mg/kg) treated group at 0, 30, 60, 90 and 120 min was 3.75 ± 0.25, 6.75 ± 0.47, 10.25 ± 0.47, 12.50 ± 0.28 and 14.25 ± 0.47 s (P < 0.001 vs. saline) respectively. The mice were pretreated with naloxone (0.5 mg/kg) and their response to A3 and B2 was measured both in acetic acid-induced writhing test and hot plate assay. The results indicated that naloxone did not reverse the action of A3 and B2 in these assays indicating A3 and B2 produce their pain relieving effect involving non opioidergic central mechanism (Figs. 3, 4, 5, 6).

### Docking of ligands with COX-1 (3N8X), COX-2 (1PXX) and human capsaicin receptor (3J9J)

**Fig. 7:**
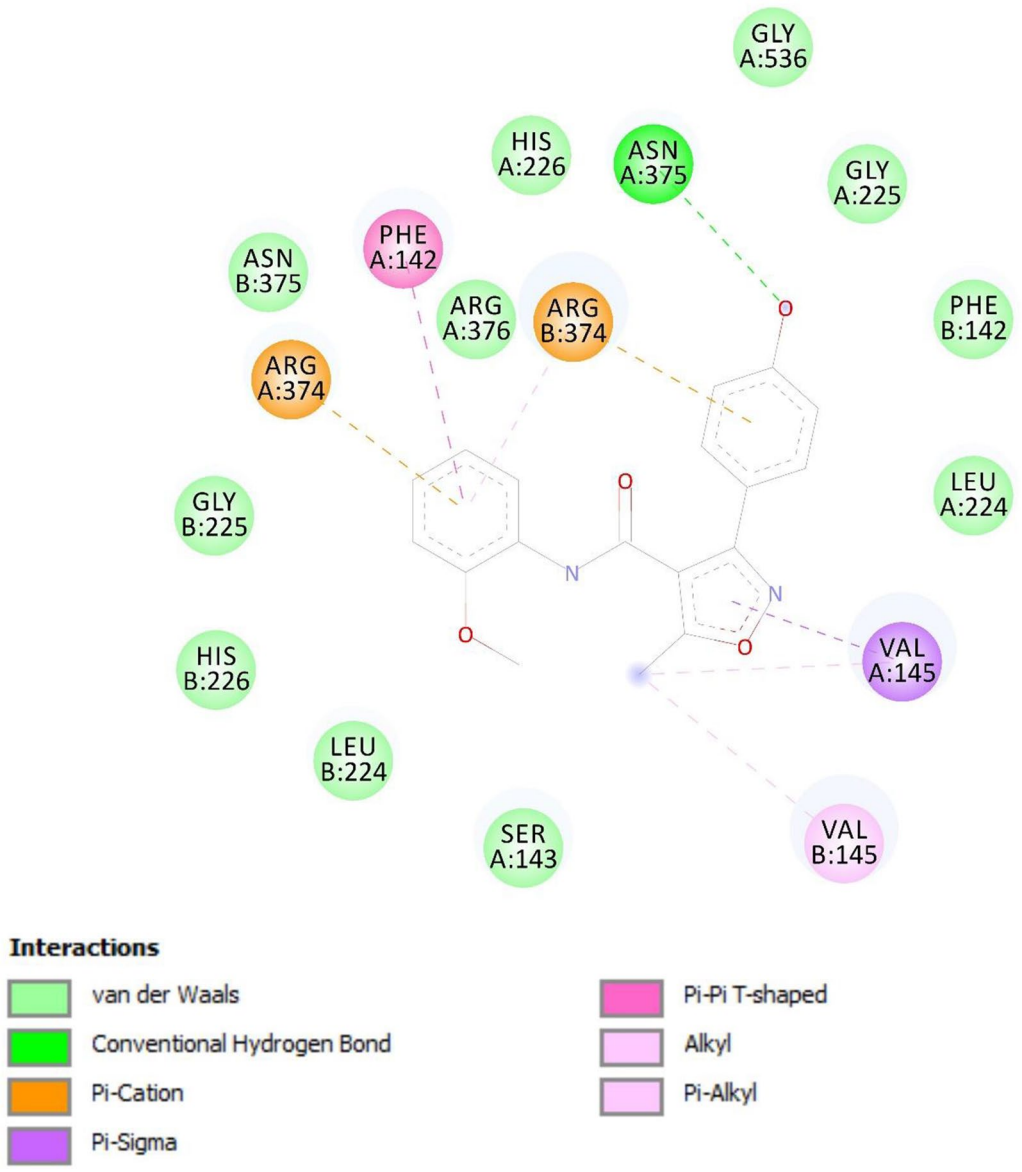
Binding interactions of compound A3 with (COX-1 3N8X)

**Fig. 8:**
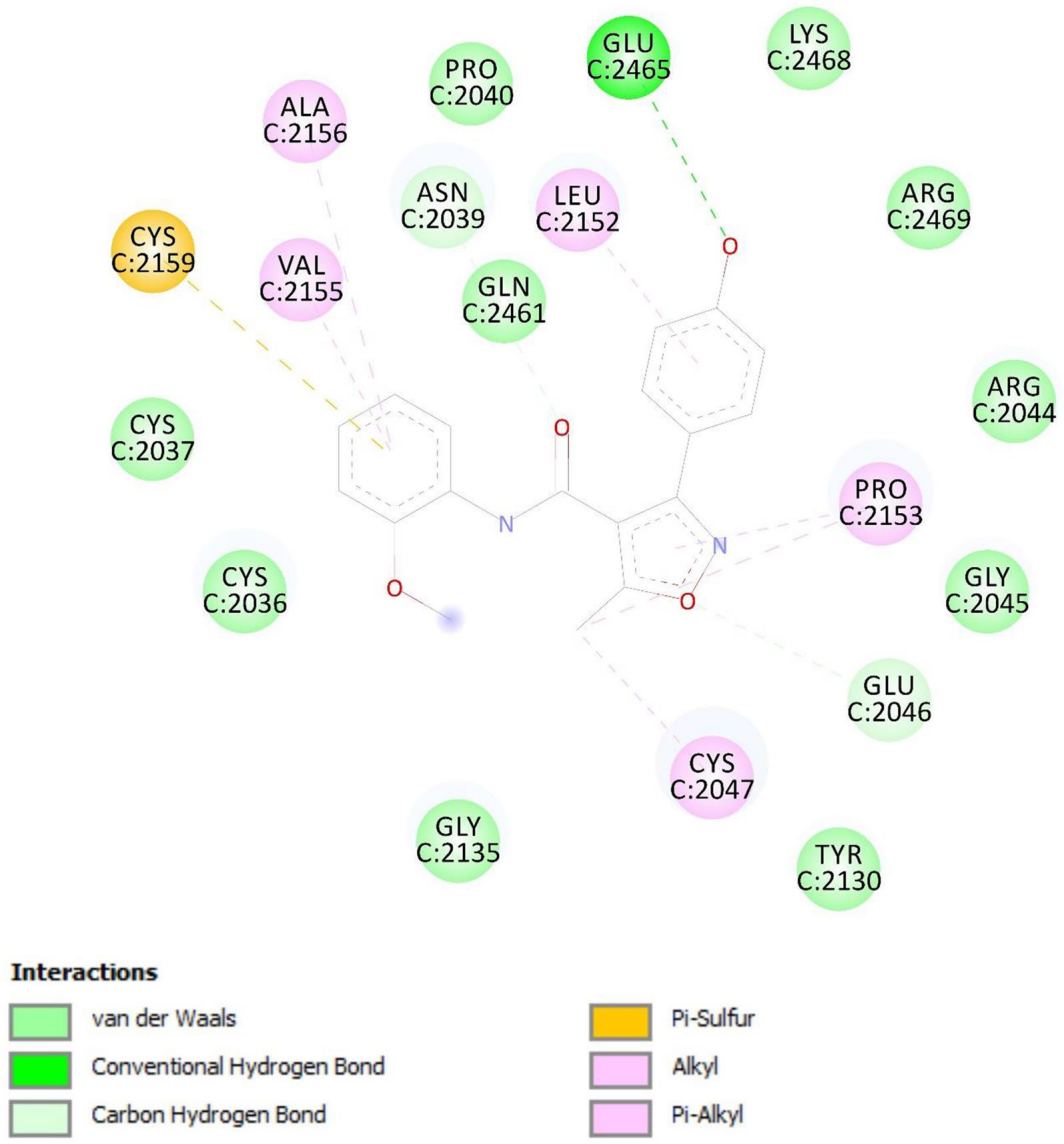
Binding interactions of compound A3 with (COX-2 1PXX)

**Fig. 9:**
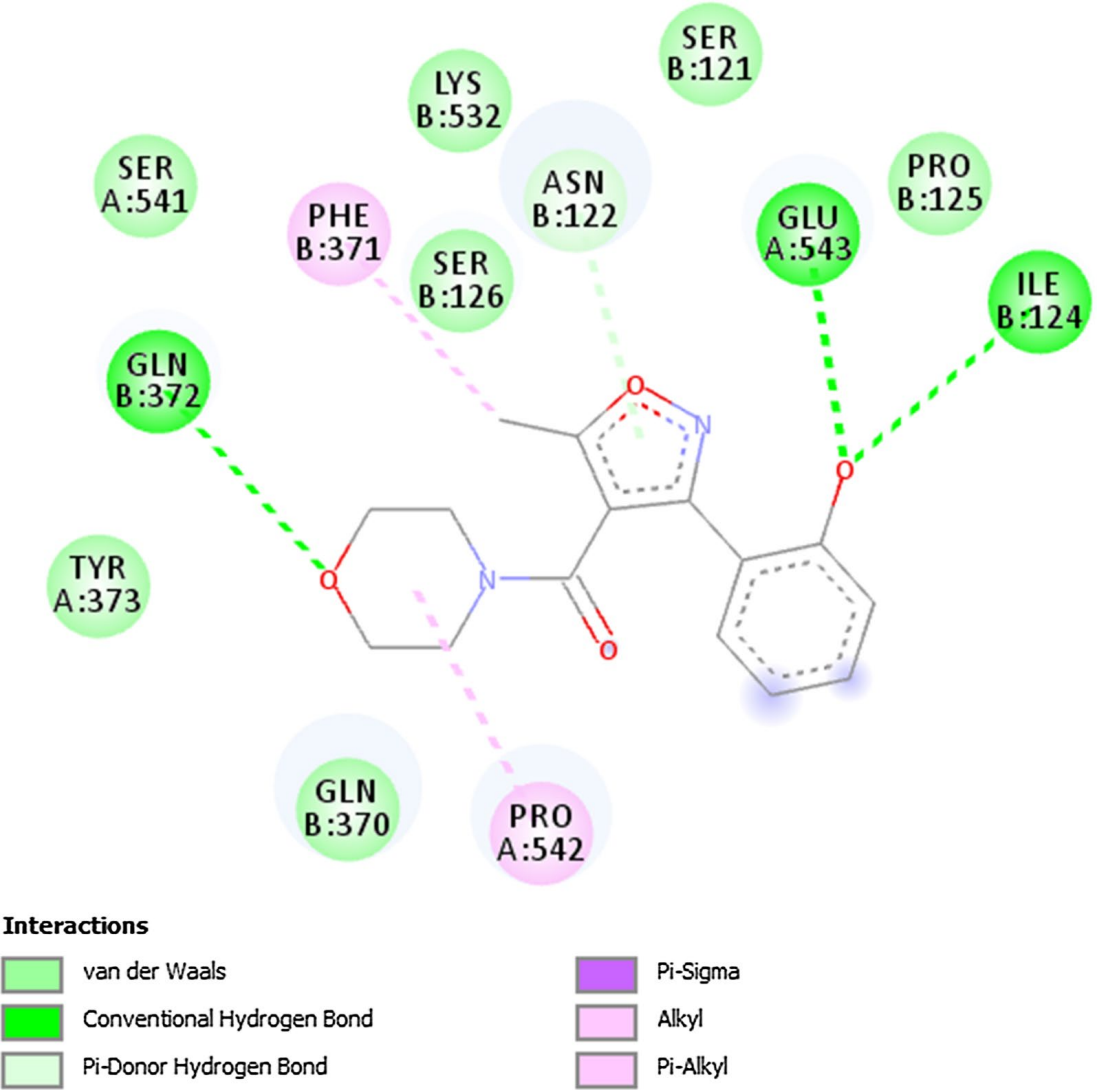
Binding interactions of compound B2 with (COX-1 3N8X)

**Fig. 10:**
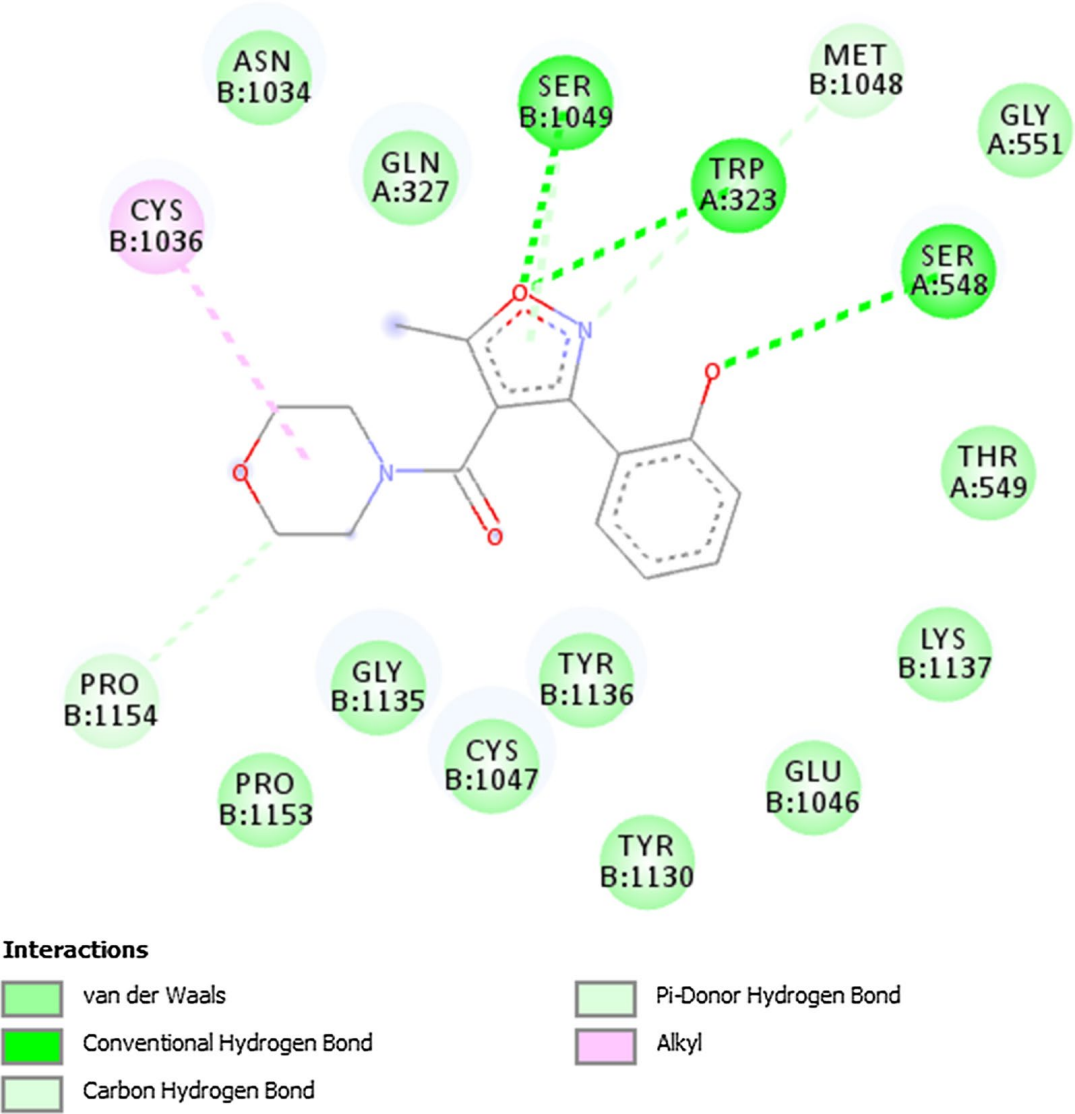
Binding interactions of compound B2 with (COX-2 1PXX)

Compounds A3 and B2 were docked against COX-1, COX-2 and human capsaicin receptor and the results obtained in the form of binding affinity (Figs. 7, 8, 9, 10 and Table 2).

**Table 2:** Docking results of A3 and B2 with COX-1, COX-2 and human capsaicin receptor

Protein targets	Ligand	Binding energy Kcal/mol
Cyclooxygenase-1 COX-1, PDB-ID: 3N8X	A3	− 8.6
	B2	− 7.9
Cyclooxygenase-2 COX-2, PDB-ID: 1PXX	A3	− 8.8
	B2	− 8.4
Human capsaicin receptor (HCR), PDB-ID: 3J9J	A3	− 7.5
	B3	− 8.2

The lowest binding energy value represents highest binding affinity. The interactions involved in binding of ligand with COX-1 3N8X, COX-2 1PXX and human capsaicin receptor HCR 3J9J were Van der Waal, conventional hydrogen bond, carbon-hydrogen bond, pi-donor hydrogen bond, pi-cation, pi-sigma, pi-anion, pi–pi t-shaped, pi-Sigma, pi-alkyl, alkyl and attractive charge. The compound A3 showed highest binding affinity with lowest binding energy with COX-2 receptor i.e., − 8.8 kcal/mol. The binding interactions involved were Van der Waal, conventional hydrogen bond, pi-donor hydrogen bond, amide pi-stacked, pi-alkyl and carbon hydrogen bond. The compounds A3 and B2 showed good binding energies with non-opioid receptors.

## Conclusion

Isoxazole is an important pharmacophore in medicinal chemistry with a wide range of pharmacological activities. Keeping in view the diverse nature of isoxazole moiety, we designed nine novel derivatives of 3-substituted-isoxazole-4-carboxamide and screened them for their analgesic potential. They showed low to moderate analgesic activity. Tramadol was used as reference standard and in all cases activity was less than the standard. Among the synthesized derivatives B2 (6 mg/kg) showed high analgesic activity as compared to tramadol, the currently used analgesic drug. Compounds A3 and B2 were docked against human non-opioid receptors (COX-1, 3N8X), (COX-2, 1PXX) and human capsaicin receptor (HCR, 3J9J) to check their affinity and analgesic potential. It is therefore concluded that our synthesized isoxazole-derivatives can be used as lead molecules for the development of new analgesic drugs with less side effects and high efficacy.

## Abbreviations

COX-1: cyclooxygenase-1 enzyme
COX-2: cyclooxygenase-2 enzyme
HCR: human capsaicin receptor
FTIR: Fourier transform infrared
H NMR: proton nuclear magnetic resonance
Ar-H: aromatic hydrogen
R_f_: relative factor
mol.: moles
Pet. Ether: petroleum ether
ppm: parts per million
